# Methylated genomic loci encoding microRNA as a biomarker panel in tissue and saliva for head and neck squamous cell carcinoma

**DOI:** 10.1186/s13148-018-0470-7

**Published:** 2018-04-03

**Authors:** Yu Cao, Katherine Green, Steve Quattlebaum, Ben Milam, Ling Lu, Dexiang Gao, Hui He, Ningning Li, Liwei Gao, Francis Hall, Matthew Whinery, Elyse Handley, Yi Ma, Tao Xu, Feng Jin, Jing Xiao, Minjie Wei, Derek Smith, Sophia Bornstein, Neil Gross, Dohun Pyeon, John Song, Shi-Long Lu

**Affiliations:** 1 Department of Otolaryngology, University of Colorado Anschutz Medical Campus, 12700 E19th Avenue, Aurora, CO 80045 USA; 2 Laboratory of Precision Oncology, China Medical University School of Pharmacy, No. 77 Puhe Road, Shenyang, 110122 China; 3 Department of Biostatistics, University of Colorado Anschutz Medical Campus, 12700 E19th Avenue, Aurora, CO 80045 USA; 4 Research Laboratory and Department of Hematology, Benxi Central Hospital, Benxi, 117000 China; 5 Department of Medical Oncology, Peking Union Medical School Hospital, Beijing, 100730 China; 6 Department of Radiation Oncology, China Japan Friendship Hospital, Beijing, China; 7 Department of Otolaryngology, The First University Hospital of China Medical University, Shenyang, 110001 China; 8 Department of Immunology and Microbiology, University of Colorado Anschutz Medical Campus, 12700 E19th Avenue, Aurora, CO 80045 USA; 9 Department of Surgical Oncology, The First University Hospital of China Medical University, Shenyang, 110001 China; 10 Department of Oral Pathology, Dental School of Dalian Medical University, Dalian, 116044 China; 11 Department of Radiation Oncology, Oregon Health & Science University, 3181 SW Sam Jackson Park Road, Portland, OR 97239 USA; 12 Department of Otolaryngology, Oregon Health & Science University, 3181 SW Sam Jackson Park Road, Portland, OR 97239 USA; 13 Department of Pathology, University of Colorado Anschutz Medical Campus, 12700 E19th Avenue, Aurora, CO 80045 USA; 14 Department of Dermatology, University of Colorado Anschutz Medical Campus, 12700 E19th Avenue, Aurora, CO 80045 USA; 15 Department of Radiation Oncology, Cornell University, New York, NY USA; 16 Department of Head and Neck Surgery, MD Anderson Cancer Center, Houston, TX USA

**Keywords:** Head and neck squamous cell carcinoma, microRNA, DNA methylation, Biomarker, Tissue, Saliva

## Abstract

**Background:**

To identify aberrant promoter methylation of genomic loci encoding microRNA (mgmiR) in head and neck squamous cell carcinoma (HNSCC) and to evaluate a biomarker panel of mgmiRs to improve the diagnostic accuracy of HNSCC in tissues and saliva.

**Methods:**

Methylation of promoter regions of mgmiR candidates was initially screened using HNSCC and control cell lines and further selected using HNSCC and control tissues by quantitative methylation-specific PCR (qMS-PCR). We then examined a panel of seven mgmiRs for validation in an expanded cohort including 189 HNSCC and 92 non-HNSCC controls. Saliva from 86 pre-treatment HNSCC patients and 108 non-HNSCC controls was also examined using this panel of seven mgmiRs to assess the potentials of clinical utilization.

**Results:**

Among the 315 screened mgmiRs, 12 mgmiRs were significantly increased in HNSCC cell lines compared to control cell lines. Seven out of the 12 mgmiRs, i.e., mgmiR9-1, mgmiR124-1, mgmiR124-2, mgmiR124-3, mgmiR129-2, mgmiR137, and mgmiR148a, were further found to significantly increase in HNSCC tumor tissues compared to control tissues. Using multivariable logistic regression with dichotomized variables, a combination of the seven mgmiRs had sensitivity and specificity of 92.6 and 92.4% in tissues and 76.7 and 86.1% in saliva, respectively. Area under the receiver operating curve for this panel was 0.97 in tissue and 0.93 in saliva. This model was validated by independent bootstrap validation and random forest analysis.

**Conclusions:**

mgmiR biomarkers represent a novel and promising screening tool, and the seven-mgmiR panel is able to robustly detect HNSCC in both patient tissue and saliva.

**Electronic supplementary material:**

The online version of this article (10.1186/s13148-018-0470-7) contains supplementary material, which is available to authorized users.

## Background

Head and neck squamous cell carcinoma (HNSCC) compromises approximately 90% of all head and neck cancers and 5% of all malignancies [1, 2]. HNSCC has also seen an increasing rate of prevalence over the past 30 years due to HPV infection [3]. Despite advancements in cancer therapy, the prognosis for HNSCC patients remains poor [3]. The low survival rate is in stark contrast to the increase in survival rates of many other cancers. One of the main reasons for the poor prognosis of HNSCC is that by the time of diagnosis, more than half of HNSCC patients have locoregionally advanced disease. Therefore, early detection may be key to improving survival rates in the future [4].

Current early screening methods for HNSCC in the clinic are limited to physical examination or optical devices by either dentists or primary care physicians. These then lead to a referral to a specialist. Around that time, medical imaging, such as MRI, CT scan, or laryngoscopy, are still the main methods for initial clinical diagnosis, leading eventually to a biopsy or surgical procedure for confirmation. [5]. These initial imaging methods are subjective, inaccurate, invasive, costly, and inconvenient. Moreover, utilizing the current system leads to significant diagnostic delays so that by the time of diagnosis, over 2/3 of HNSCC patients already have in advanced stage disease [6]. Thus, development of an objective, accurate, non-invasive, low cost, and convenient method for early detection would be highly beneficial.

Development of cancer-specific biomarkers for detection of initial HNSCC and recurrence has been widely explored using DNA-based (loss of heterozygosity, mutation, and DNA methylation), RNA-based, and protein-based assays in both patient tissues and saliva [7–17]. DNA methylation usually occurs early in the process of tumorigenesis and has been widely developed as a basis for biomarkers for human cancers [18–21]. Several methylation markers have been approved by the FDA for clinical application, including *MGMT* in glioblastoma [22] and the ColoGuard stool-based screen for colorectal cancer patients [23]. Development of DNA methylation biomarkers for HNSCC detection has also been reported [7, 19]. However, biomarker studies are still limited to the research phase, and no biomarker-based assays for early detection have been used clinically for HNSCC patients.

MicroRNAs (miRNAs) are a class of non-coding small RNAs, which negatively regulate gene expression at the post-transcriptional level [24]. A number of miRNAs have been found to be deregulated in human cancers through various mechanisms [25]. One of the mechanisms responsible for reduced or loss of miRNA expression is epigenetic silencing of miRNA genes by DNA methylation at the genomic loci encoding the miRNAs [26]. Currently, more than 20 miRNAs have been reported to be silenced by DNA methylation in multiple human cancers [27, 28]. We have reported that DNA methylation of miR9 specifically occurred in a subset of human HNSCC tissue samples [29]. However, their potential as a novel class of biomarker for human cancer detection has not been fully assessed.

We hypothesized that DNA methylation at the genomic loci encoding miRNA (mgmiRs) represents a novel class of modification and could be efficiently utilized for human cancer including HNSCC. We tested this hypothesis by screening and selecting mgmiRs in both cell lines and tissues from HNSCC patients and normal controls. We then investigated panel of seven mgmiRs, i.e., 9-1, 124-1, 124-2, 124-3, 129-2, 137, and 148a, in an expanded patient’s cohort, including 189 HNSCC tissues and 92 control tissues. The translational application of this panel of mgmiRs was further evaluated in saliva from 86 HNSCC patients and 108 control patients. Lastly, we assessed the association of individual mgmiRs with demographic and clinical pathological information in both tissue and saliva.

## Methods

### Patient information

This study was conducted on human HNSCC surgical samples from both the University of Colorado Anschutz Medical Campus and the Oregon Health & Science University (OHSU) under the Institutional Review Board approval protocols from each institution. A written informed consent was obtained from each subject. A total of 281 different tissue specimens were used. Among them, 189 samples were HNSCC specimens from the time of surgical resection and constituted our “tumor” group. Ninety-two samples were from non-HNSCC patients undergoing surgeries for sleep apnea or tonsillectomy with no history of malignancy and used as our “non-HNSCC control” group (Table 1). Saliva samples were collected in 86 previously untreated HNSCC patients and 108 control patients including subjects enrolled in a community screening study and non-HNSCC patients undergoing surgeries for sleep apnea or tonsillectomy (Table 1). Enrollment included collection of demographic information, risk factor history, and clinical pathological information. All information was registered in a Research Electronic Data Capture (REDCap) database.

**Table 1:** Demographic and clinicopathological information of participated patients

Variables	Tissue			Saliva		
	Controls (*n* = 92)	HNSCC (*n* = 189)	*p*	Controls (*n* = 108)	HNSCC n = (86)	*p*
Age (years, mean ± SD)	54.98 ± 17.63	63.23 ± 11.58	< 0.0001	53.77 ± 16.04	61.07 ± 9.66	0.0001
Gender			0.38			0.20
Male (%)	65.2%(60/92)	70.1% (131/187)		69.4% (75/108)	77.9% (67/86)	
Female (%)	34.8%(32/92)	29.9% (56/187)		30.6% (33/108)	22.1% (19/86)	
Smoking status			0.35			0.25
Never	25.6% (23/90)	22.4% (37/165)		26.9% (29/108)	19.8% (17/86)	
Current	21.1% (19/90)	30.3% (50/165)		16.7% (18/108)	24.4% (21/86)	
Ex-smoker	53.3%(48/90)	47.3% (78//165)		56.5% (61/108)	55.8% (48/86)	
Location						
OSCC	n/a	50.8% (95/187)		n/a	50.6% (42/83)	
OPSCC	n/a	23.5% (44/187)		n/a	33.7% (28/83)	
LSCC	n/a	25.7% (48/187)		n/a	15.7% (13/83)	
HPV status						
Positive	n/a	37.9% (25/66)		n/a	n/a	
Negative	n/a	62.1% (41/66)		n/a	n/a	
Tumor size						
T1	n/a	24.7% (46/186)		n/a	25.6% (20/78)	
T2	n/a	26.3% (49/186)		n/a	33.3% (26/78)	
T3	n/a	22.1% (41/186)		n/a	14.1% (11/78)	
T4	n/a	26.9% (50/186)		n/a	26.9% (21/78)	
Node status						
N0	n/a	41.9% (78/186)		n/a	36.8% (28/76)	
N1	n/a	19.9% (37/186)		n/a	7.9% (6/76)	
N2	n/a	35.5% (66/186)		n/a	52.6% (40/76)	
N3	n/a	2.7% (5/186)		n/a	2.6% (2/76)	
Stage						
I	n/a	18.3%(34/186)		n/a	13.2%(10/76)	
II	n/a	10.8%(20/186)		n/a	17.1%(13/76)	
III	n/a	31.7%(59/186)		n/a	22.4%(17/76)	
IV	n/a	39.3%(73/186)		n/a	47.4%(36/76)	

*n/a* not available

### Preparation of samples

After harvesting, tissue was immediately taken to the laboratory where it was frozen and stored in liquid nitrogen until DNA extraction. For saliva collection, patients/volunteers were required to refrain from eating, drinking, chewing gums, and smoking 30 min before head. At the time of saliva collection, patients gargled with normal saline solution two times for around 20 s each time. Patients/volunteers were then instructed to spit their saliva into the collection tube for 5 min without swallowing. Once collected, samples were immediately frozen and stored at − 80 °C until ready for use.

### DNA extraction and bisulfite conversion of genomic DNA

Tissue DNA was extracted from each tissue sample using the DNeasy Blood & Tissue kit (QIAGEN, Hilden, Germany), and saliva DNA was extracted using the QiaAmp DNA mini kit (QIAGEN, Hilden, Germany) following the manufacturer’s instructions. Quantity and quality of the extracted genomic DNAs were measured using the Nanovue spectrophotometer (GE Healthcare). Bisulfite conversion of 1 μg genomic DNA was performed as described in the EZ DNA Methylation-Gold kit (Zymo, Irvine, CA, USA) to create a template for qMS-PCR. The bisulfate-modified genomic DNA was resuspended in 100 μl of water and stored at − 80 °C.

### Quantitative methylation-specific PCR

Bisulfite-treated DNA was then used as a template for qMS-PCR, which was performed using the methylation-specific primers. For the primer designs, genomic sequence for each miRNAs including 1000 base upstream were obtained from the UCSC genomic browser website. The primers for methylation analysis were designed on the basis of this sequence using MethPrimer software. All primer sequences are available upon request. The analysis was performed using quantitative methylation-specific PCR (qMS-PCR).

For each individual marker, the qMS-PCR protocol was optimized prior to running the samples, in order to identify the proper annealing temperature and maximize the results to obtain a typical sigmoid result curve. Melting curves and gel were integrated to determine the specificity of each marker. Variables were adjusted for the temperature, number of cycles, and length of each cycle. Each reaction was performed in a 20-μl PCR mixture consisting of 2 μl of bisulfite-converted DNA, 5 nmol/L of forward primer, 5 nmol/L of reverse primer, and 4 μl SYBR-green supermix (Biorad, Hercules, CA, USA). QMS-PCR was run in triplicate on the CFX connect™ real-time detection system (Biorad, Hercules, USA). First, samples were denatured at 95 °C for 5 min, this was followed by 40 cycles of 95 °C for 30 s, and lastly for a given primer, samples were exposed to the optimized annealing/extension temperature for 1 min. Standardization was done by using UMSCC10A cells and subjecting the cells to methylation in vitro with excess Sss1 methyltransferase (New England Biolabs, Ipswich, MA, USA) to generate a completely methylated DNA, and serial dilutions of this DNA were used for constructing the calibration curve for each plate. Water and reaction mix were also included in each plate to serve as negative controls and to ensure that there was no contamination. For each sample within each marker, a relative methylation level was calculated using the difference in Ct values by the standard 2^−ΔCt^ method in which β-actin was used as an internal reference gene. A Ct of 15 to 30 was considered a high methylation level, and a Ct of 35, a low methylation level. A Ct value of more than 40 was considered as undetectable.

### HPV detection

An HPV signal was detected with amplification of L1 consensus sequence using primers GP5+/GP6+ as previously described [30]. Briefly, 20 ng of genomic DNA extracted from tissue samples and cell lines was used for PCR amplification by the forward primer GP5+ (5′-TTTGTTACTGTGGTAGATACTAC) and the reverse primer GP6+ (5′-GAAAAATAAACTGTAAATCATATTC). The final PCR products were analyzed by 2% agarose gel electrophoresis and ethidium bromide staining.

### Statistical analysis

Descriptive statistics such as mean, standard deviation, proportion, and percentage were used to summarize demographic and clinicopathological characteristics of study subjects. Chi-square or two-group *T* tests, as appropriate, were used to compare HNSCC patients and control patients.

A univariate and combined analysis of the seven selected mgmiRs was carried out using logistic regression. The receiver operating curve (ROC) was computed for each of the analyses. For each of the univariate analyses, the Youden’s index (J) was used to determine the optimal cutoff point to dichotomize a continuous mgmiR based on the ROC plots. J can be formally defined as J = Maximum (sensitivity + specificity − 1) on a ROC plot. The cut-point that archives this maximum is referred to as the optimal cut-point because it optimizes the biomarker’s differentiating ability when equal weight is given to sensitivity and specificity [31]. Additionally, to indicate diagnostic accuracy of each mgmiR, sensitivity, specificity, positive predictive values (PPVs), and negative predictive values (NPVs) were provided. The same quantities were also calculated for the combination of the seven mgmiRs.

The bootstrap method was used to validate the performance of the combination of the seven mgmiRs internally, where 2000 random samples with replacement were generated with each sample containing the same number of observations as the original dataset. The same combined analysis of the seven selected mgmiRs was carried out on each sample and the average AUC and its 95% confidence interval (2.5 and 97.5 percentiles) across the 2000 samples were reported. In addition, random forest, a nonparametric classification approach was used to estimate the prediction performance using the seven mgmiRs using the CARET package in R (Kuhn M. 2016. R package version 6.0-73). Random forest analyses were conducted separately for tissue and saliva samples. The dataset was split into training and testing datasets, using a 2/3 to 1/3 split and random sampling. The random forest models were trained using 5000 trees and 1000 bootstrap resamples. In addition, for each type of sample (i.e., tissue or saliva), it was assessed whether the presence of demographic information (i.e., age, gender, and smoking history) improved prediction results. The random forest models were then applied to the testing dataset, and the ROC and area under the ROC (AUC) were determined. Youden’s index was used to determine a cutoff point on the ROC, and sensitivity and specificity were determined. The median and its 95% confidence interval for the sensitivity and specificity were estimated using 2000 bootstrap resamples using the pROC package in R.

## Results

### DNA methylation at genomic loci encoding microRNAs were identified in HNSCC cell lines

We utilized the following approaches to screen candidate methylated genomic loci encoding miRNAs (mgmiRs) in HNSCC: (1) The UCSC genome browser was used to obtain 1 Kb genomic sequences [GRch38] of 5′-UTRs of 315 primary (Pri-) miRNAs in the *Homo sapiens* miRBase [from has-let-7a-1 to has-mir-499b in miRbase, http://www.mirbase.org]. (2) The CpG island prediction software (MethPrimer) was then utilized to identify 26 genomic loci with CpG islands (defined as island size > 200 bp, GC content > 50%, observed/expectation > 0.6). (3) By designing methylation-specific primers and running quantitative methylation-specific PCR (qMS-PCR), we amplified methylation signals in 22 mgmiRs and found 12 mgmiRs that had increased methylation in human HNSCC cell lines compared to normal head and neck cell lines (Additional file 1: Figures S1 and S2). As shown in Additional file 2: Table S1, we examined relative methylation levels in 12 HNSCC cell lines including cell lines derived from age (ranging from 22 to 70 years), both male and female, human papillomavirus (HPV)-positive and HPV-negative HNSCCs, an HNSCC from Fanconi anemia patient, and different anatomic sites of the head and neck region. Four head and neck normal cell lines were included as controls.

### HNSCC patients and control patients were recruited for this study

We included 189 tumor tissues from HNSCC patients and 92 control tissues from non-HNSCC patients undergoing surgeries for sleep apnea or tonsillectomy (Table 1). Clinical and demographic variables were similar in cases and controls with exception of age. Subjects with HNSCC patients were older than controls (63.23 vs. 54.98 years). Primary tumor sites included oral squamous cell carcinoma (OSCC), 95 cases; oropharynx SCC (OPSCC), 44 cases; larynx SCC (LSCC), 48 cases; and two cases without location information. Among the 66 HNSCC tissue samples tested for HPV status, there were 25 (37.9%) HPV-positive cases and 41 (62.1%) HPV-negative cases. Pathologic stage at diagnosis was T1 in 46 cases, T2 in 49 cases, T3 in 41 cases, and T4 in 50 cases; and N0 in 78 cases, N1 in 37 cases, N2 in 66 cases, and N3 in five cases. Clinical staging was stage I in 34 cases, II in 20 cases, III in 59 cases, and IV in 73 cases. There were three HNSCC cases without TNM information (Table 1). We also included 86 saliva from pre-treatment HNSCC patients and 108 from control patients of either undergoing surgeries for sleep apnea or tonsillectomy, or coming for community screening. Similar to tissue samples, clinical and demographic variables were similar in cases and controls with exception of age. Subjects with HNSCC patients were older than controls (61.07 vs. 53.77 years). Primary tumor sites included OSCC, 42 cases; OPSCC, 28 cases; LSCC, 13 cases, and three cases without location information. Pathologic stage at diagnosis was T1 in 20 cases, T2 in 26 cases, T3 in 11 cases, T4 in 21 cases, and eight cases without information of tumor size; and N0 in 28 cases, N1 in 6 cases, N2 in 40 cases, and N3 in two cases. Clinical staging was stage I in 10 cases, II in 13 cases, III in 17 cases, and IV in 36 cases. There are ten HNSCC cases without node and stage information (Table 1).

### A panel of seven mgmiR biomarker was identified and validated in HNSCC patient tissues

To further identify mgmiRs which can distinguish HNSCC from control patient samples, we first examined the 12 mgmiRs in a small sample cohort including 30 HNSCC patients’ tissue samples, and 25 age and gender-matched normal head and neck tissues from either tonsillitis or sleep apnea patients. Based on the comparison of relative methylation levels, seven mgmiRs, i.e., 9-1, 124-1, 124-2, 124-3, 129, 137, and 148a, showed significant elevation in HNSCC tissue compared to non-HNSCC control tissues and were selected for further study (Additional file 1: Figure S3).

**Fig. 1:**
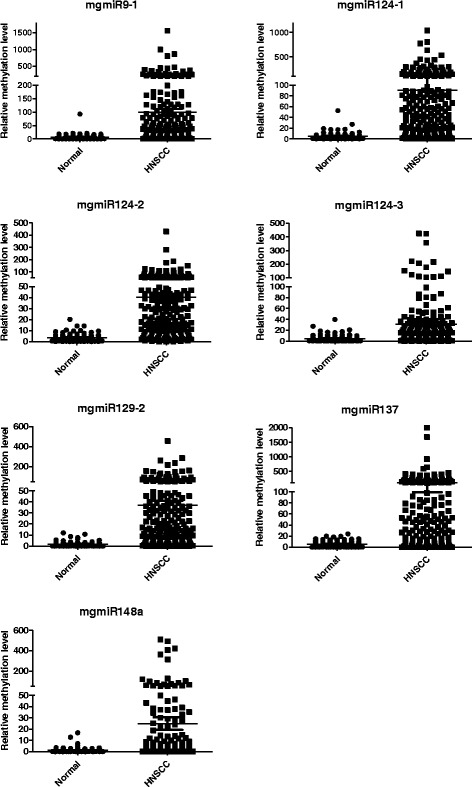
Relative methylation levels for seven mgmiRs (9-1, 124-1, 124-2, 124-3, 129-2, 137, 148a) in tissues from 189 HNSCC patients and 92 controls. The quantity of methylated mgmiRs was expressed as fold changes from the methylated mgmiR to that from the reference gene β-actin

We then expanded examination of the seven mgmiRs to additional 159 HNSCC and 67 non-HNSCC control cases. Fig. 1 and Additional file 2: Table S2 show the relative methylation levels of the seven mgmiRs in the total of 189 (30 + 159) HNSCC and 86 (25 + 67) non-HNSCC control tissues. The total methylation level in the HNSCC group was significantly higher (*p* < 0.0001) than that in the control group for all the seven mgmiRs. All data was analyzed using both dichotomized and continuous variables. ROC curves for HNSCC detection were generated using either dichotomized variables or continuous variables (Additional file 1: Figure S4A for each mgmiR). As shown in Table 2, the univariate assessment of HNSCC diagnosis from each of the seven dichotomized mgmiR variables in tissue had sensitivities and specificities ranging from 43.9 to 77.3% and 85.9 to 100%, respectively. However, the combined assessment of the seven mgmiRs in tissue resulted in a higher sensitivity and a specificity that was within the range seen in the univariate assessment, 92.6 and 92.4%, respectively. The combined assessment of the seven mgmiRs yielded 92.5% accuracy, 96.2% PPV, and 85.9% NPV with an area under curve (AUC) of 0.97 (Table 2). Since age difference is a confounder factor, we also included age together with the seven mgmiRs for the combined assessment. As shown in Table 2, inclusion of age slightly enhanced specificity but no significant changes with other parameters. Internal bootstrap validation had AUC of 0.97 with a 95% CI of 0.95–0.99. The results using continuous forms of the mgmiR variables as opposed to dichotomous forms were similar with 93.6% sensitivity, 92.4% specificity, and AUC of 0.98 (Additional file 2: Table S3).

**Table 2:** Univariate and multivariable logistic regression with dichotomized variables in tissues

mgmiRs	Sensitivity	Specificity	Accuracy	PPV	NPV	AUC
mgmiR9-1	65.6	85.9	72.2	90.5	54.9	0.76
mgmiR124-1	72.0	93.5	79.0	95.8	61.9	0.83
mgmiR124-2	77.3	96.7	83.6	98.0	67.4	0.87
mgmiR124-3	49.7	92.4	63.7	93.1	47.2	0.71
mgmiR129-2	73.5	94.6	80.4	96.5	63.5	0.84
mgmiR137	61.4	100	74.0	100	55.8	0.81
mgmiR148a	43.9	91.3	59.4	91.2	44.2	0.61
Combined^1^	92.6	92.4	92.5	96.2	85.9	0.97
Combined^2^	92.1	94.6	92.9	97.2	85.3	0.97

^1^The combined model includes all the seven mgmiRs

^2^The combined model includes all the seven mgmiRs and age

### MgmiRs were associated with HPV infection and could be detected in early cancer stage HNSCC tissues

We looked for association between either individual mgmiR or in combination and clinical pathological characteristics. For the mgmiR combination, we first estimated the probability of a tissue being estimated as positive using logistic regression with the seven mgmiRs then assessed the association between a characteristic and the estimated status (case or control) of the tissue. In general, the associations between the mgmiRs and location of HNSCC, smoking status, tumor size, node status, and stage were stronger for the combination of the seven mgmiRs compared to their individual assessments (Table 3). Notably, mgmiR124-2 and mgmiR129-2 were found to detect significantly more HPV-positive than HPV-negative HNSCC cases (88.0 vs. 65.9%, *p* = 0.04; 84.0 vs. 56.1%, *p* = 0.02, respectively). There were no significant associations between tumor size, stage, and percentage of positive cases detected by either individual mgmiR or the seven mgmiRs as a panel. However, most importantly, 93.5% of T1 and 91.2% of stage I HNSCC can be detected by the seven mgmiRs as a panel (Table 3). Similarly, there were no significant associations between node status (N0 vs. N+) with the exception of mgmiR124-1, which detected more N0 cases than N+ cases (79.5 vs. 65.7%, *p* = 0.04, Table 3).

**Table 3:** Association between mgmiRs and clinicopathological characteristics in HNSCC tissues

	mgmiR9-1 %(*n*)	mgmiR124-1 %(*n*)	mgmiR124-2 %(*n*)	mgmiR124-3 %(*n*)	mgmiR129-2 %(*n*)	mgmiR137 %(*n*)	mgmiR148a %(*n*)	Combined mgmiRs %(*n*)
Location								
OSCC (95)	66.3(63)	72.6(69)	71.6(68)	52.6(50)	73.7(70)	64.2(61)	43.2(41)	92.6(88)
OPSCC (44)	75(33)	72.7(32)	86.4(38)	50(22)	79.6(35)	61.4(27)	40.9(18)	97.7(43)
LSCC (48)	56.3(27)	68.8(33)	81.3(39)	43.8(21)	68.8(33)	58.3(28)	50.0(24)	89.6(43)
Smoking								
Never (37)	70.3(26)	67.6(25)	73.0(27)	56.8(21)	64.9(24)	59.5(22)	51.4(19)	89.2(33)
Current (50)	74.0(37)	76.0(38)	86.0(43)	44.0(22)	74.0(37)	64.0(32)	36.0(18)	96.0(48)
Ex (78)	64.1(50)	75.6(59)	73.1(57)	53.9(42)	78.2(61)	68.0(53)	46.2(36)	96.2(75)
HPV								
Positive (25)	68.0(17)	68.0(17)	88(22)	72(18)	84.0(21)	52.0(13)	48.0(12)	96(24)
Negative (41)	51.2(21)	63.4(26)	65.9(27)*	53.7(22)	56.1(23)*	61.0(25)	39.0(16)	80.5(33)*
Tumor size								
T1 (46)	76.0(35)	69.6(32)	65.2(30)	56.5(26)	56.5(26)	63.0(29)	34.8(16)	93.5(43)
T2 (49)	65.3(32)	77.6(38)	87.8(43)	51.0(25)	83.7(41)	65.3(32)	53.1(26)	95.9(47)
T3 (41)	58.5(24)	58.5(24)	75.6(31)	39.0(16)	70.7(29)	48.8(20)	36.6(15)	87.8(36)
T4 (50)	62.0(31)	78.0(39)	80.0(40)	52.0(26)	82.0(41)	68.0(34)	50.0(25)	94.0(47)
Node status								
N0 (78)	69.2(54)	79.5(62)*	76.9(60)	56.4(44)	69.2(54)	60.3(47)	42.3(33)	94.9(74)
N+ (108)	63.0(68)	65.7(71)	77.8(84)	45.4(49)	76.9(83)	63.0(68)	45.4(49)	91.7(99)
Stage								
I (34)	73.5(25)	73.5(25)	64.7(22)	55.9(19)	55.9(19)	58.8(20)	32.4(11)	91.2(31)
II (20)	55.0(11)	80.0(16)	85.0(17)	55.0(11)	80.0(16)	75.0(15)	55.0(11)	95.0(19)
III (59)	67.8(40)	66.1(39)	74.6(44)	44.7(24)	72.9(43)	59.3(35)	37.3(22)	91.5(54)
IV (73)	63.0(46)	72.6(53)	83.6(61)	53.4(39)	80.8(59)	61.6(45)	52.1(38)	94.5(69)

*p < 0.05

### The seven mgmiR biomarkers were validated in HNSCC patient saliva

**Fig. 2:**
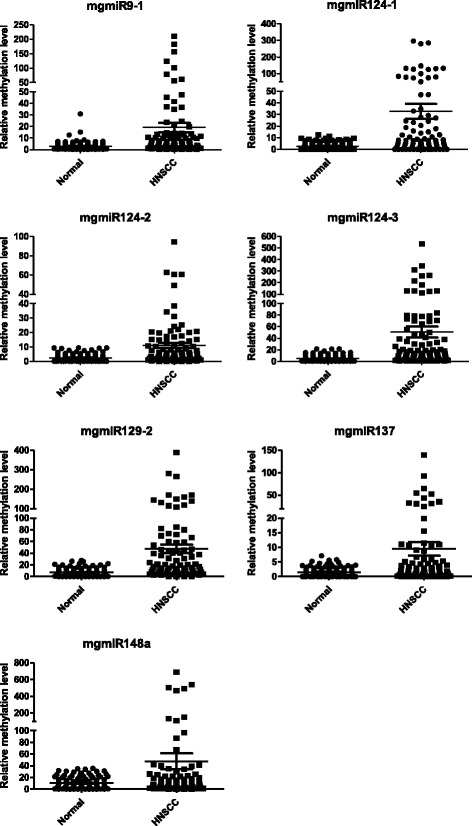
Relative methylation levels for the seven mgmiRs (9-1, 124-1, 124-2, 124-3, 129-2, 137, 148a) in saliva from 86 HNSCC patients and 108 controls. The quantity of methylated mgmiRs was expressed as fold changes from the methylated mgmiR to that from the reference gene β-actin

We then examined the seven mgmiR biomarkers in saliva from 86 HNSCC patients and 108 normal controls. The relative methylation levels of the seven mgmiRs in HNSCC and control saliva are shown in Fig. 2 and Additional file 2: Table S4. The total methylation level in the HNSCC group was significantly higher than the control group across the seven mgmiRs. All data were analyzed using both dichotomized and continuous variables. ROC curves for HNSCC detection were generated using either dichotomized variables or continuous variables for each mgmiR (Additional file 1: Figure S4B). The sensitivity and specificity using dichotomized variables for HNSCC diagnosis from single mgmiR in saliva ranged from 19.8 to 72.1% and 74.1 to 97.2%, respectively, and from the seven combined mgmiRs in saliva 76.7 and 86.1%, respectively. The combined seven mgmiRs yielded 83.0% accuracy, 83.5% PPV, and 82.6% NPV with AUC 0.93 (Table 4). Inclusion of age together with the seven mgmiRs slightly increased specificity (Table 4). Internal bootstrap validation had AUC of 0.93 with a 95% CI of 0.89–0.96. The results using continuous variables were better to those using dichotomized variables with 83.7% sensitivity, 95.4% specificity, and AUC of 0.95 (Additional file 2: Table S5).

**Table 4:** Univariate and multivariable logistic regression with dichotomized variables in saliva

mgmiRs	Sensitivity	Specificity	Accuracy	PPV	NPV	AUC
mgmiR9-1	48.8	93.5	73.7	85.7	69.7	0.71
mgmiR124-1	66. 3	74.1	70.6	67.1	73.4	0.70
mgmiR124-2	45.4	88.9	69.6	76.5	67.1	0.67
mgmiR124-3	53.5	96.3	77.3	92.0	72.2	0.75
mgmiR129-2	72.1	74.1	73.2	68.9	76.9	0.73
mgmiR137	33.7	94.4	67.5	82.9	64.2	0.64
mgmiR148a	19.8	97.2	62.9	85	60.3	0.58
Combined^1^	76.7	86.1	82.0	81.5	82.3	0.92
Combined^2^	76.7	88.0	83.0	83.5	82.6	0.93

^1^The combined model includes all the seven mgmiRs

^2^The combined model includes all the seven mgmiRs and age

### MgmiR was detected in the saliva of early HNSCC

Similar to the tissue sample results, the associations between the mgmiRs and location of HNSCC, smoking status, tumor size, node status, and stage were generally stronger for the combination of the seven mgmiRs compared to their individual assessments (Table 5). The combination of seven mgmiRs can detect 71.4, 82.1, and 76.9% of OSCC, OPSCC, and LSCC, respectively. However, there was no significant association between tumor location and positive cases detected by mgmiR either individually or in combination (Table 5). Importantly, 85.0% of T1 HNSCC and 80.0% of stage I HNSCCs could be detected by the panel of mgmiRs, although there were no significant associations between tumor size, node status, stage, and mgmiR-positive cases detected by either individual mgmiR or the combination (Table 5).

**Table 5:** Association between mgmiRs and clinicopathologcal characteristics in HNSCC saliva

	mgmiR9-1 %(*n*)	mgmiR124-1 %(*n*)	mgmiR124-2 %(*n*)	mgmiR124-3 %(*n*)	mgmiR129-2 %(*n*)	mgmiR137 %(*n*)	mgmiR148a %(*n*)	Combined mgmiRs %(*n*)
Location								
OSCC (42)	40.5(17)	69.1(29)	45.2(19)	40.5(17)	73.8(31)	31.0(13)	26.2(11)	71.4(30)
OPSCC (28)	57.1(16)	57.1(16)	46.4(13)	64.3(18)	67.9(19)	32.1(9)	17.9(5)	82.1(23)
LSCC (13)	53.9(7)	84.6(11)	46.2(6)	61.5(8)	69.2(9)	46.2(6)	7.70(1)	76.9(10)
Smoking								
Never (17)	64.7(11)	70.6(12)	52.9(9)	58.8(10)	64.7(11)	35.3(6)	11.8(2)	82.4(14)
Current (21)	52.4(11)	66.7(14)	28.6(6)	57.1(12)	81.0(17)	19.1(4)	23.8(5)	76.2(16)
Ex (48)	41.7(20)	64.6(31)	50.0(24)	50.0(24)	70.8(34)	39.6(19)	20.8(10)	75.0(36)
Tumor size								
T1 (20)	55.0(11)	65.0(13)	30.0(6)	55.0(11)	75.0(15)	35.0(7)	30.0(6)	85.0(17)
T2 (26)	38.5(10)	61.5(16)	57.7(15)	42.3(11)	73.1(19)	34.6(9)	19.2(5)	73.1(19)
T3 (11)	45.5(5)	72.7(8)	45.5(5)	63.6(7)	72.7(8)	45.5(5)	18.2(2)	63.6(7)
T4 (21)	42.9(9)	76.2(16)	47.6(10)	47.6(10)	61.9(13)	28.6(6)	14.3(3)	71.4(15)
Node status								
N0 (28)	57.1(16)	67.9(19)	39.3(11)	50.0(14)	75.0(21)	32.1(9)	14.3(4)	75.0(21)
N+ (48)	35.4(17)	68.8(33)	52.1(25)	47.9(23)	66.7(32)	37.5(18)	25.0(12)	72.9(35)
Stage								
I (10)	60.0(6)	80.0 (8)	30.0(3)	40.0(4)	80.0(8)	40.0(4)	20.0(2)	80.0(8)
II (13)	46.2(6)	53.9(7)	53.9(7)	53.9(7)	76.9(10)	23.1(3)	23.1(3)	69.2(9)
III (17)	47.1(8)	52.9(9)	41.2(7)	52.9(9)	64.7(11)	41.2(7)	11.8(2)	76.5(13)
IV (36)	36.1(13)	77.8(28)	52.8(19)	47.2(17)	66.7(24)	36.1(13)	25.0 (9)	72.2(26)

### Complementary prediction accuracy and performance assessment further validated the panel of mgmiR biomarkers in HNSCC

The mgmiR biomarkers were further evaluated for tissue and saliva samples using random forest models. Similar to the combined logistic regression model results reported above, the random forest models included all seven mgmiR biomarkers. In addition, demographic information’s influence (age, gender, and smoking status) on prediction performance was also assessed. Excluding demographic information, the randomly selected tissue training dataset had 186 subjects, 62 (33%) controls and 124 (67%) cancers, and the independent testing dataset had 92 subjects, 30 (33%) controls and 62 (67%) cancers. With the inclusion of demographic information, the tissue training dataset had 170 subjects, 60 (35%) controls and 110 (65%) cancers, and the testing dataset had 84 subjects, 30 (36%) controls and 54 (64%) cancers, due to missing in demographic variables. Alternatively, no demographic information was missing for the saliva samples and therefore the training and testing datasets were the same between models with and without demographic information. The saliva training dataset had 130 subjects, 72 (55%) controls and 58 (45%) cancers, and the testing dataset had 64 subjects, 36 (56%) controls and 28 (44%) cancers. Model performance (i.e., AUC, sensitivity, and specificity) using the random forest approach was similar to the combined seven-mgmiR logistic regression results for both the tissue and saliva samples when demographic information was not included (Table 6). The mean decrease in Gini index was used to evaluate variable importance. The demographic variables were found to be the least important variables in both the tissue and saliva samples (Additional file 1: Figures S5-S6). However, including demographic information in the random forest models resulted in slightly but not significantly higher AUCs, sensitivities, and specificities (Table 6).

**Table 6:** Random forest model results for tissue and saliva samples using either mgmiRs and demographic variables or the mgmiRs alone

Model	AUC % (95% CI)	Sensitivity % [median (2.5–97.5%)]	Specificity % [median (2.5–97.5%)]	PPV	NPV
Tissue					
mgmiRs and demographics	98.9 (97.1–100)	96.3 (90.7-100)	96.7 (90.0-100)	98.2	93.8
mgmiRs	99.4 (98.6–100)	93.7 (87.3–98.4)	100 (100–100)	100	88.2
Saliva					
mgmiRs and demographics	93.8 (87.3–100)	85.7 (71.4–96.4)	94.4 (86.1–100)	93.3	90.0
mgmiRs	93.5 (87.2–99.7)	78.6 (60.7–92.9)	100 (100–100)	100	85.7

Univariate and multivariable logistic regression with dichotomized variables

## Discussion

Early detection of cancers in high-risk populations has increased patient survival rates, which is exemplified in colonoscopy for colon cancer, pap smear for cervical cancer, and mammogram for breast cancer [32]. Recently, the FDA approved the use of a stool-DNA test for colon cancer as a screening tool. This stool-DNA test, which can be used ahead of colonoscopy, significantly improved sensitivity and specificity of colon cancer detection, patient compliance, and reduced costs [23]. Nearly all current diagnostic methods for HNSCC in clinic rely on physical examination, medical imaging, and endoscopy techniques. Molecular tests for HNSCC have been widely explored [7, 19]. However, their application in clinic has been limited by their poor performance (i.e., low sensitivity and specificity). In this study, we reported a high sensitivity and specificity molecular test can be achieved by examining DNA methylation on the genomic loci of microRNA (mgmiR). This is the first study to evaluate the effectiveness of mgmiR, a novel class of molecule, as a biomarker panel for HNSCC diagnosis using tissue and saliva samples from a large HNSCC population.

Our “mgmiR” technique combines several advantages of different molecular test technologies: (1) It is a genomic DNA-based technique. Analysis of RNA or protein in saliva samples relies on the stability of the mRNA or protein, which is always a challenge as saliva harbors high levels of RNAse and proteases [33, 34]. In contrast, genomic DNA has a higher stability and is less susceptible to be affected by storage and shipping than RNA- or protein-based technologies. (2) It is a methylation-based technique. In contrast with examining expression level of mRNA, miRNA itself [35], or protein, which can be either positive (increased) or negative (decreased), DNA methylation converts a negative signal (reduced or loss of expression) into a positive signal, which can be used for the detection of cancer-specific signal in a high background of normal non-cancer cells. In contrast with detecting mutations, which usually occur at multiple sites in individual tumors. DNA methylation usually occurs on the same region of a gene, such as the promoter, which greatly simplifies design and interpretation of screening tests. In addition, DNA methylation occurs at the early stage of cancer development, providing the opportunity for early detection of cancer [19]. It also has tissue specificity, providing a foundation for detection of tumor of unknown primary, which is exemplified in a EPICUP clinical trial study [36–38]. (3) Instead of detecting methylation of coding genes, mgmiR detects genomic loci encoding for microRNA. Given the lower numbers of miRNAs, compared to coding mRNAs, mgmiR-based technique will compensate or at least provide novel biomarkers for current DNA methylation-based HNSCC detection. (4) It is a qPCR-based technique which has been more widely accepted in clinical laboratories. It is highly sensitive, simple, easier to handle, and has a lower cost than mutation search using next-generation sequencing (NGS).

While the development of “liquid biopsy” is almost focused on the analysis of cell-free DNA and/or circulating tumor cells in the blood, saliva, together with sputum, urine, etc., constitutes major resources for molecular testing using “body fluids.” Blood-based liquid biopsy has been largely applied to patients with late-stage cancers or serves as companion biomarkers for treatment. The sensitivity and specificity may limit their applicability for detection of early-stage cancers. For example, saliva has been shown to be a more sensitive predictor than blood for detection of HNSCC [17]. Using the recently developed most sensitive NGS, only about 50% of colon cancer patients can be detected at stage I, in comparison with ~ 90% of patients that can be detected at stages II–IV [39]. Although we do not have data from blood to directly compare with the data from saliva, the sensitivity from saliva for stage I HNSCC patients in our study is 80%, which is higher than the results from the blood reported in the literature [39]. Thus, it is likely that the saliva-based method is more sensitive and can detect cancer earlier than blood-based method. In addition, for screening purposes, the nature of non-invasive, self-sampling, convenient, and low-cost of saliva test make it a better screening tool particularly for rural or community primary care settings [40].

While this report focuses on identification and validation of a panel of mgmiRs as biomarkers for HNSCC by rigorous statistical analysis in a large sample size, the functions and mechanism of individual mgmiRs and corresponding miRNA in HNSCC pathogenesis are not included in this report. One interesting question is how the miRNAs are regulated by their mgmiRs. We have reported that miR9 expression is restored upon DNA demethylation agent 5-aza-cytidine treatment [29], and similar results have been obtained for miR124, miR137, miR129-2, and miR148a (manuscripts in preparation), indicating DNA methylation on mgmiRs is a common mechanism for silencing miRNA expression. Another interesting mechanism is how each individual miRNA functions in HNSCC pathogenesis. While detailed functional analysis including identification of their downstream target genes is beyond the scope of this report, we published the tumor suppression role of miR9 on cell proliferation [29] and are preparing separate manuscripts on characterizing functional role of individual miRNA and their target genes in HNSCC pathogenesis.

Our controls are comprised of a significant numbers of tonsillitis patients; however, we have shown inflammation does not affect the ability of mgmiRs in distinguishing cancer and controls, suggesting this panel of mgmiRs is cancer-specific. An interesting focus of study is why mgmiRs are methylated in cancer but not in normal tissue. We do not quite understand the underlying mechanism yet, but reports on aberrant regulation of DNA methytransferases (DNMTs) by either smoking or HPV could be potential mechanisms [41, 42]. We have not tested these mgmiRs in other cancers. However, there are reports of mgmi124-2 detection in cervical cancers [43]. Interestingly, both cervical and HNSCC are squamous cell carcinomas, and HPV is the common etiological factor in both cervical and HPV+ HNSCC patients. Whether this marker represents a common marker for HPV-related squamous cell carcinoma is a potential area of future investigation.

The association between mgmiR biomarker(s) and clinical pathological data yield several interesting findings: (1) Statistical analysis showed no significant difference of positive cases detected by mgmiRs in tumor size, node involvement, and tumor stage. More importantly, T1 size tumors were detected in 93.5% of tissues and 85.0% of saliva samples, N0 tumors were detected in 94.9% of tissues and 75% of saliva samples, and stage I HNSCC patients were detected in 91.2% of tissues and 80.0% of saliva samples. These data clearly demonstrate the clinical usefulness of using the mgmiR panel for early detection of HNSCC. (2) Although the positive percentages of LSCC detected by either mgmiR or the seven mgmiRs as a panel were lower than OSCC and OPSCC tumor tissues, it is not statistically significant. However, mgmiR148a seems to be limited in its ability to detect LSCC, and it may be possible to use the remaining six mgmiRs for LSCC detection in saliva. One question here is the age difference between the control group and HNSCC group would affect methylation of mgmiRs in general population, since aging has been shown to affect both methylation and cancer development [44, 45]. We thus include age as a confounder with the seven mgmiRs and compare prediction performance with and without age (Tables 2 and 4). We have also performed a random forest model of the seven mgmiRs together with age, gender, and smoking status (Table 6). Age itself is shown to slightly contribute to the prediction and is ranked lowest in comparison with the other seven mgmiRs. Anyway, it would be ideal in the future to include more aged-matched controls whenever it is possible in clinical study.

While the incidence of HPV-negative HNSCC gradually drops down due to cessation of smoking, the incidence of HPV-positive HNSCC has increased more than twofold in the past 20 years [46]. In our data, smoking status does not affect the results of the assay. However, the mgmiR124-2 and mgmiR129-2 detected more HPV-positive HNSCC cases than HPV-negative HNSCC cases. Unfortunately, we do not have HPV data on non-HNSCC control patients at this moment, which would help us to better distinguish if this association is HPV-associated or HPV-cancer-associated, and this warrants a future study. One of the problems of current HPV virus detection for either HNSCC or cervical cancer is that the high number of patients who are HPV positive without having a disease. Detection of HPV virus alone cannot discriminate truly oncogenic infection from transient “bystander” infection. Immunostaining of the surrogate marker p16 on tissue sections frequently generates false-positive results [47]. Thus, similar to the triage strategy used in cervical cancer [48], the detection of mgmiRs in saliva samples will be useful to further separate high-risk oncogenic infectious HPV-positive patients from low-risk “passenger” HPV infection and have great promise as triage tool for HPV-positive HNSCC diagnostics.

## Conclusions

We have successfully developed a diagnostic panel using mgmiRs markers that demonstrated superior sensitivity and specificity in the detection of HNSCC in both tissues and saliva. The high sensitivity in detection of T1, N0, and stage I HNSCC of this panel of markers suggests their values in the early detection of HNSCC. Further assessment using pre-malignant lesions and prospectively monitoring high-risk patients will measure their usefulness in clinic. The quantitative nature makes the panel of mgmiRs an ideal candidate to use in surveillance of HNSCC patients, including treatment efficacy, prognosis, and prediction of recurrence and metastasis. Evaluation of these clinical applications using this panel of mgmiR markers are undergoing.

## Additional files

Additional file 1: **Figure S1.** Flow chart of mgmiRs search for HNSCC. **Figure S2.** Screening of mgmiR in HNSCC using HNSCC and control cell lines. Relative methylation level of the mgmiRs examined by qMS-PCR in 12 HNSCC cell lines (HNSCC) and 4 head and neck control cell lines (Normal). Red frames highlight mgmiRs with significance difference between HNSCCs and normal (*p* < 0.05). **Figure S3.** Selection of mgmiR in HNSCC using HNSCC and control tissues. Relative methylation level of the mgmiRs examined by qMS-PCR in 30 HNSCC tissues (HNSCC) and 25 control tissues (Normal). Red frames highlight mgmiRs with significance difference between HNSCCs and normal (*p* < 0.05). **Figure S4.** ROC curves using continuous variables for HNSCC detection. (**A**). ROC curves comparing the seven mgmiRs with the largest areas under the curve for tissues. (**B**). ROC curves comparing the seven mgmiRs with the largest areas under the curve for saliva. **Figure S5.** Variable importance plot from the Random Forest analysis for tissue data including (**A**) or excluding (**B**) demographic information. **Figure S6.** Variable importance plot from the Random Forest analysis for saliva data including (**A**) or excluding (**B**) demographic information. (ZIP 509 kb)

Additional file 2: **Table S1.** Relative methylation levels of mgmiRs in human HNSCC cell lines and normal head and neck cell lines. **Table S2.** Relative methylation level in tissues from 189 HNSCC patients and 92 normal controls. **Table S3.** Univariate and multivariable logistic regression with continuous variables in tissues. **Table S4.** Relative methylation level in saliva from 86 HNSCC patients and 108 normal controls. **Table S5.** Univariate and multivariable logistic regression with continuous variables in saliva. (DOCX 42 kb)

## Abbreviations

AUC: Area under the ROC
HNSCC: Head and neck squamous cell carcinoma
HPV: Human papillomavirus
LSCC: Larynx SCC
mgmiR: Methylation of genomic loci encoding microRNA
miRNA: MicroRNA
NPV: Negative predictive value
OPSCC: Oropharynx SCC
OSCC: Oral squamous cell carcinoma
PPV: Positive predictive value
qMS-PCR: Quantitative methylation-specific PCR
ROC: Receiver operating curve
