# Supplementary material for: Methylated genomic loci encoding microRNA as a biomarker panel in tissue and saliva for head and neck squamous cell carcinoma
Source: Clin Epigenetics. 2018 Apr 3;10:43. doi: 10.1186/s13148-018-0470-7 (PMC5883341; doi:10.1186/s13148-018-0470-7)
Supplement: Supplementary file 1 — Figure S1. Flow chart of mgmiRs search for HNSCC. Figure S2. Screening of mgmiR in HNSCC using HNSCC and control cell lines. Relative methylation level of the mgmiRs examined by qMS-PCR in 12 HNSCC cell lines (HNSCC) and 4 head and neck control cell lines (Normal). Red frames highlight mgmiRs with significance difference between HNSCCs and normal (p < 0.05). Figure S3. Selection of mgmiR in HNSCC using HNSCC and control tissues. Relative methylation level of the mgmiRs examined by qMS-PCR in 30 HNSCC tissues (HNSCC) and 25 control tissues (Normal). Red frames highlight mgmiRs with significance difference between HNSCCs and normal (p < 0.05). Figure S4. ROC curves using continuous variables for HNSCC detection. (A). ROC curves comparing the seven mgmiRs with the largest areas under the curve for tissues. (B). ROC curves comparing the seven mgmiRs with the largest areas under the curve for saliva. Figure S5. Variable importance plot from the Random Forest analysis for tissue data including (A) or excluding (B) demographic information. Figure S6. Variable importance plot from the Random Forest analysis for saliva data including (A) or excluding (B) demographic information. (ZIP 509 kb) [file 13148_2018_470_MOESM1_ESM.zip › mgmiR-supple-revision_021118.docx]

Methylated Genomic Loci Encoding microRNA as a Biomarker Panel in Tissue and Saliva for Head and Neck Squamous Cell Carcinoma

Additional file 1: Figure S1. Flow chart of mgmiRs search for HNSCC.

Figure S2. Screening of mgmiR in HNSCC using HNSCC and control cell lines. Relative methylation level of the mgmiRs examined by qMS-PCR in 12 HNSCC cell lines (HNSCC) and 4 head and neck control cell lines (Normal). Red frames highlight mgmiRs with significance difference between HNSCCs and normal (p<0.05).

Figure S3. Selection of mgmiR in HNSCC using HNSCC and control tissues. Relative methylation level of the mgmiRs examined by qMS-PCR in 30 HNSCC tissues (HNSCC) and 25 control tissues (Normal). Red frames highlight mgmiRs with significance difference between HNSCCs and normal (p<0.05).

Figure S4. ROC curves using continuous variables for HNSCC detection. (A). ROC curves comparing the seven mgmiRs with the largest areas under the curve for tissues. (B). ROC curves comparing the seven mgmiRs with the largest areas under the curve for saliva.

Figure S5. Variable importance plot from the Random Forest analysis for tissue data including (A) or excluding (B) demographic information.

Figure S6. Variable importance plot from the Random Forest analysis for saliva data including (A) or excluding (B) demographic information.
