# Supplementary material for: Methylated genomic loci encoding microRNA as a biomarker panel in tissue and saliva for head and neck squamous cell carcinoma
Source: Clin Epigenetics. 2018 Apr 3;10:43. doi: 10.1186/s13148-018-0470-7 (PMC5883341; doi:10.1186/s13148-018-0470-7)
Supplement: Supplementary file 2 — Table S1. Relative methylation levels of mgmiRs in human HNSCC cell lines and normal head and neck cell lines. Table S2. Relative methylation level in tissues from 189 HNSCC patients and 92 normal controls. Table S3. Univariate and multivariable logistic regression with continuous variables in tissues. Table S4. Relative methylation level in saliva from 86 HNSCC patients and 108 normal controls. Table S5. Univariate and multivariable logistic regression with continuous variables in saliva. (DOCX 42 kb) [file 13148_2018_470_MOESM2_ESM.docx]

Methylated Genomic Loci Encoding microRNA as a Biomarker Panel in Tissue and Saliva for Head and Neck Squamous Cell Carcinoma

Additional file 1: Figure S1. Flow chart of mgmiRs search for HNSCC.

Additional file 2: Figure S2. Screening of mgmiR in HNSCC using HNSCC and control cell lines. Relative methylation level of the mgmiRs examined by qMS-PCR in 12 HNSCC cell lines (HNSCC) and 4 head and neck control cell lines (Normal). Red frames highlight mgmiRs with significance difference between HNSCCs and normal (p<0.05).

Additional file 4: Figure S3. Selection of mgmiR in HNSCC using HNSCC and control tissues. Relative methylation level of the mgmiRs examined by qMS-PCR in 30 HNSCC tissues (HNSCC) and 25 control tissues (Normal). Red frames highlight mgmiRs with significance difference between HNSCCs and normal (p<0.05).

Additional file 6: Figure S4. ROC curves using continuous variables for HNSCC detection. (A). ROC curves comparing the seven mgmiRs with the largest areas under the curve for tissues. (B). ROC curves comparing the seven mgmiRs with the largest areas under the curve for saliva.

Additional file 10: Figure S5. Variable importance plot from the Random Forest analysis for tissue data including (A) or excluding (B) demographic information.

Additional file 11: Figure S6. Variable importance plot from the Random Forest analysis for saliva data including (A) or excluding (B) demographic information.

### Additional file 3: Table S1. Relative methylation levels of mgmiRs in human HNSCC cell lines and normal head and neck cell lines.

| Cell lines | Sources | 9-1  (Chr.1) | 124-1  (Chr.8) | 124-2  (Chr.8) | 124-3  (Chr.20) | 129-2 (Chr.11) | 137  (Chr.1) | 148a  (Chr.7) |  |
| --- | --- | --- | --- | --- | --- | --- | --- | --- | --- |
| UMSCC10A | LSCC | 2.5 | 5.3 | 26.8 | 102.7 | 16.6 | 8.3 | 1 |  |
| UMSCC10B | Ln mets | 0 | 236.4 | 64.4 | 105.4 | 73.2 | 55.9 | 25.9 |  |
| UMSCC22A | HPSCC | 20.5 | 0 | 52.6 | 215.1 | 37.4 | 25.5 | 9.3 |  |
| UMSCC22B | Ln mets | 50.6 | 287.3 | 100.3 | 304.4 | 24.7 | 28.9 | 25.7 |  |
| UMSCC2 | OSCC rec. | 86.3 | 104.3 | 96.0 | 155.8 | 0 | 14.7 | 61.2 |  |
| UMSCC47 | OSCC,HPV+ | 18.0 | 254.3 | 123.9 | 202.0 | 0 | 0 | 102.9 |  |
| FaDu | PSCC | 140.3 | 533.0 | 223.0 | 388.9 | 46.6 | 188.7 | 218.9 |  |
| Cal27 | OSCC | 53.9 | 101.2 | 135.5 | 442.4 | 22.2 | 43.8 | 35.3 |  |
| SCC9 | OSCC | 4.6 | 0 | 10.9 | 123.7 | 68.6 | 2.5 | 199.6 |  |
| SCC25 | OSCC | 0.6 | 7.4 | 68.5 | 31.0 | 16.0 | 73.6 | 46.1 |  |
| Detroit562 | PSCC | 8.3 | 133.4 | 29.9 | 140.3 | 58.6 | 27.3 | 62.7 |  |
| Vu1365 | OSCC, FA-A | 22.7 | 120.5 | 167.2 | 490.8 | 59.0 | 72.5 | 95.3 |  |
| OKF6 | Keratinocytes | 1 | 1 | 1 | 1 | 0 | 0 | 0 |  |
| NHEK | Keratinocytes | 0 | 0.3 | 0.9 | 0.3 | 0 | 0 | 0 |  |
| NIKS | Keratinocytes | 9.0 | 0.3 | 0.2 | 0.5 | 1 | 12.5 | 0 |  |
| Hacat | Keratinocytes | 14.5 | 26.5 | 0.8 | 0 | 0 | 7.8 | 0 |  |

LSCC: laryngeal SCC, Ln mets: lymph node metastasis, HPSCC: hypopharynx SCC, OSCC rec: oral SCC recurrence, HPV: human papilloma virus, PSCC: pharynx SCC, FA-A: Fanconi anemia A

Additional file 5: Table S2. Relative methylation level in tissues from 189 HNSCC patients and 92 normal controls

|  | **normal** | **HNSCC** | ***p*** |
| --- | --- | --- | --- |
| n | 92 | 189 |  |
| mgmiR9-1 | 7.12±1.10 | 98.61±13.05 | <0.0001 |
| mgmiR124-1 | 4.87±0.72 | 90.46±10.54 | <0.0001 |
| mgmiR124-2 | 3.59±0.37 | 40.32±3.43 | <0.0001 |
| mgmiR124-3 | 4.75±0.67 | 31.38±4.45 | <0.0001 |
| mgmiR129-2 | 1.75±0.24 | 36.88±4.10 | <0.0001 |
| mgmiR137 | 5.62±0.58 | 115.2±16.14 | <0.0001 |
| mgmiR148a | 1.14±0.25 | 25.00±5.51 | <0.0001 |

Additional file 7: Table S3. Univariate and multivariable logistic regression with continuous variables in tissues

|  | Sensitivity | Specificity | accuracy | PPV | NPV | AUC |
| --- | --- | --- | --- | --- | --- | --- |
| mgmiR9-1 | 75.7 | 65.2 | 72.2 | 81.7 | 56.6 | 0.79 |
| mgmiR124-1 | 78.8 | 85.9 | 81.0 | 92.0 | 66.4 | 0.86 |
| mgmiR124-2 | 83.1 | 85.9 | 84.0 | 92.4 | 71.2 | 0.90 |
| mgmiR124-3 | 79.9 | 52.2 | 70.8 | 77.4 | 55.8 | 0.77 |
| mgmiR129-2 | 80.4 | 87.0 | 82.6 | 92.7 | 68.4 | 0.88 |
| mgmiR137 | 73.5 | 71.7 | 73.0 | 84.2 | 56.9 | 0.81 |
| mgmiR148a | 57.1 | 72.8 | 62.3 | 81.2 | 42.3 | 0.72 |
| Combined^1^ | 92.1 | 97.8 | 94.0 | 98.9 | 85.7 | 0.96 |
| Combined^2^ | 93.6 | 92.4 | 93.2 | 96.1 | 87.6 | 0.98 |

1. The combined model includes all the 7 mgmiRs above in the table
2. The combined model includes all the 7 mgmiRs above in the table and age.

Additional file 8: Table S4. Relative methylation level in saliva from 86 HNSCC patients and 108 normal controls

|  | **controls** | **HNSCC** | **p** |
| --- | --- | --- | --- |
| n | 108 | 86 |  |
| mgmiR9-1 | 2.75±0.36 | 19.22±4.12 | 0.0001 |
| mgmiR124-1 | 2.71±0.29 | 32.73±6.59 | <0.0001 |
| mgmiR124-2 | 2.43±0.24 | 11.08±1.77 | <0.0001 |
| mgmiR124-3 | 4.63±0.50 | 50.70±9.47 | <0.0001 |
| mgmiR129-2 | 7.07±0.63 | 47.75±7.28 | <0.0001 |
| mgmiR137 | 1.48±0.15 | 9.52±2.30 | 0.0008 |
| mgmiR148a | 10.51±0.95 | 47.48±13.71 | 0.0086 |

Additional file 9: Table S5. Univariate and multivariable logistic regression with continuous variables in saliva

|  | Sensitivity | Specificity | accuracy | PPV | NPV | AUC |
| --- | --- | --- | --- | --- | --- | --- |
| mgmiR9-1 | 48.8 | 93.5 | 73.7 | 85.7 | 69.7 | 0.74 |
| mgmiR124-1 | 66.3 | 70.1 | 70.6 | 67.1 | 73.4 | 0.72 |
| mgmiR124-2 | 45.4 | 88.9 | 69.6 | 76.5 | 67.1 | 0.72 |
| mgmiR124-3 | 53.5 | 96.3 | 77.3 | 92.0 | 72.2 | 0.76 |
| mgmiR129-2 | 72.1 | 74.1 | 73.2 | 68.9 | 76.9 | 0.76 |
| mgmiR137 | 33.7 | 94.4.0 | 67.5 | 82.9 | 64.2 | 0.66 |
| mgmiR148a | 19.8 | 97.2 | 62.9 | 85.0 | 60.3 | 0.54 |
| Combined^1^ | 84.9 | 95.4 | 90.2 | 93.6 | 88.8 | 0.95 |
| Combined^2^ | 83.7 | 95.4 | 90.2 | 93.5 | 88.0 | 0.95 |

1. The combined model includes all the 7 mgmiRs above in the table
2. The combined model includes all the 7 mgmiRs above in the table and age.

^1^
